# Supplementary material for: Heightened TLR7/9-Induced IL-10 and CXCL13 Production with Dysregulated NF-ҝB Activation in CD11chiCD11b+ Dendritic Cells in NZB/W F1 Mice
Source: Int J Mol Sci. 2019 Sep 19;20(18):4639. doi: 10.3390/ijms20184639 (PMC6770860; doi:10.3390/ijms20184639)
Supplement: Supplementary file 1 [file ijms-20-04639-s001.zip › ijms-580326-SI.pdf]

Supplementary Materials:

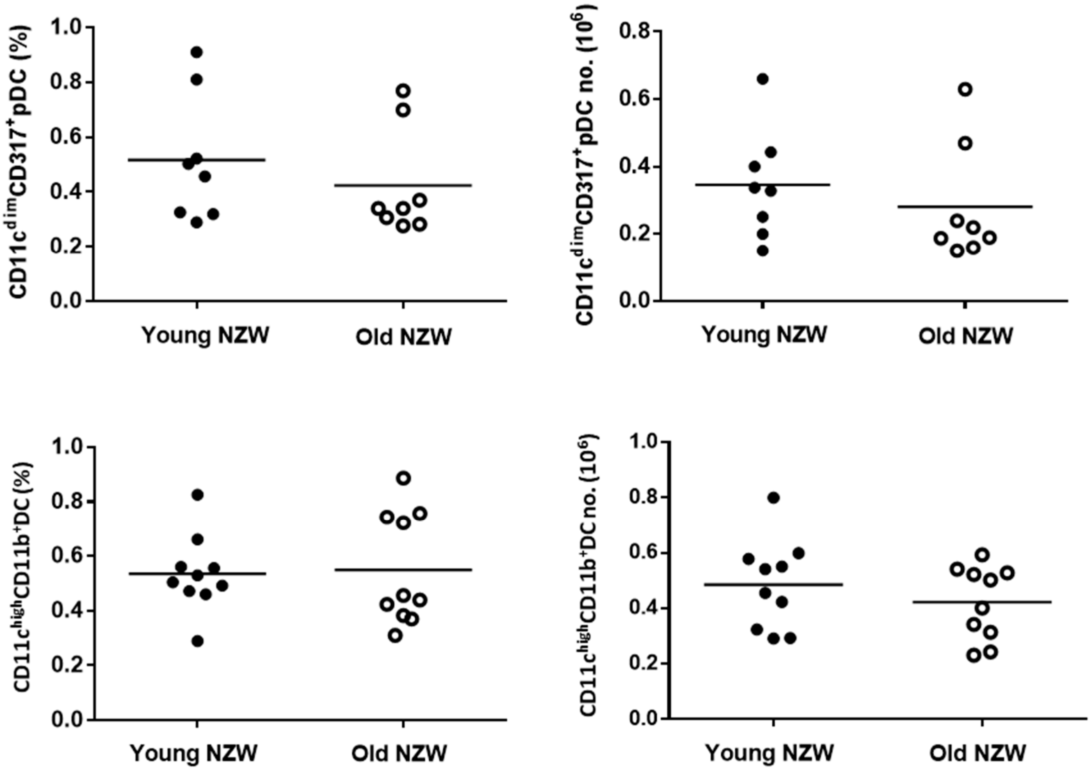

Supplementary Figure 1. Abundance of pDC and CD11c^{hi}CD11b^{+} DCs in young and old NZW control. The abundance of splenic pDC and CD11c^{hi}CD11b^{+} DCs in NZW was evaluated separately and were age- and sex-matched to experimental BWF1. Total splenocytes from NZW were stained with the pDC and mDC markers CD11c, CD11b and CD317. Summary plots comparing the frequency and total number of splenic CD11c^{dim}CD317^{+}pDC and CD11c^{hi}CD11b^{+} DCs are shown. Each symbol represents an individual mouse and Student's *t*-test was used for statistical analysis.

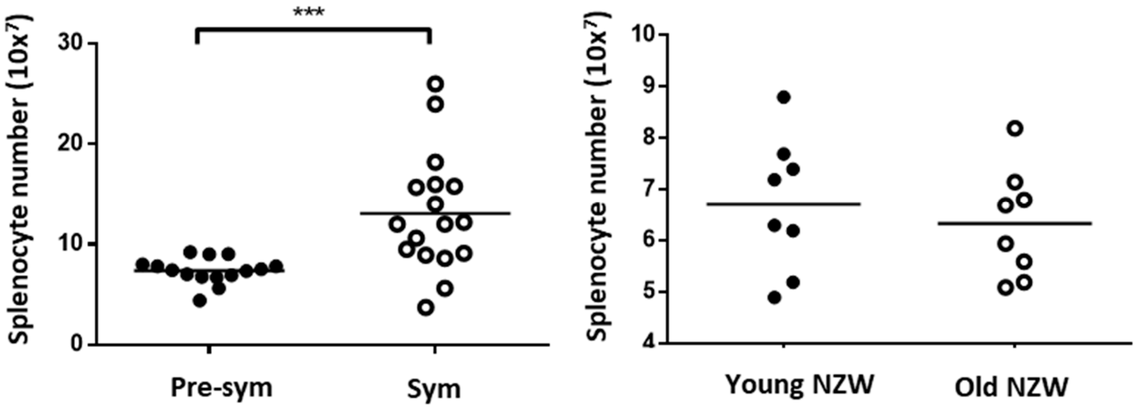

Supplementary Figure 2. Total number of splenocytes in BWF1 and NZW at different stages. Splenic cellularity of BWF1 pre-symptomatic and symptomatic mice were evaluated by determining the total number of splenocytes. The amount of splenocytes was quantified by counting. NZW was evaluated separately and were age- and sex-matched to experimental BWF1 as control. Student's *t*-test was used for statistical analysis (*** $p \leq 0.001$) and each symbol represents an individual mouse.

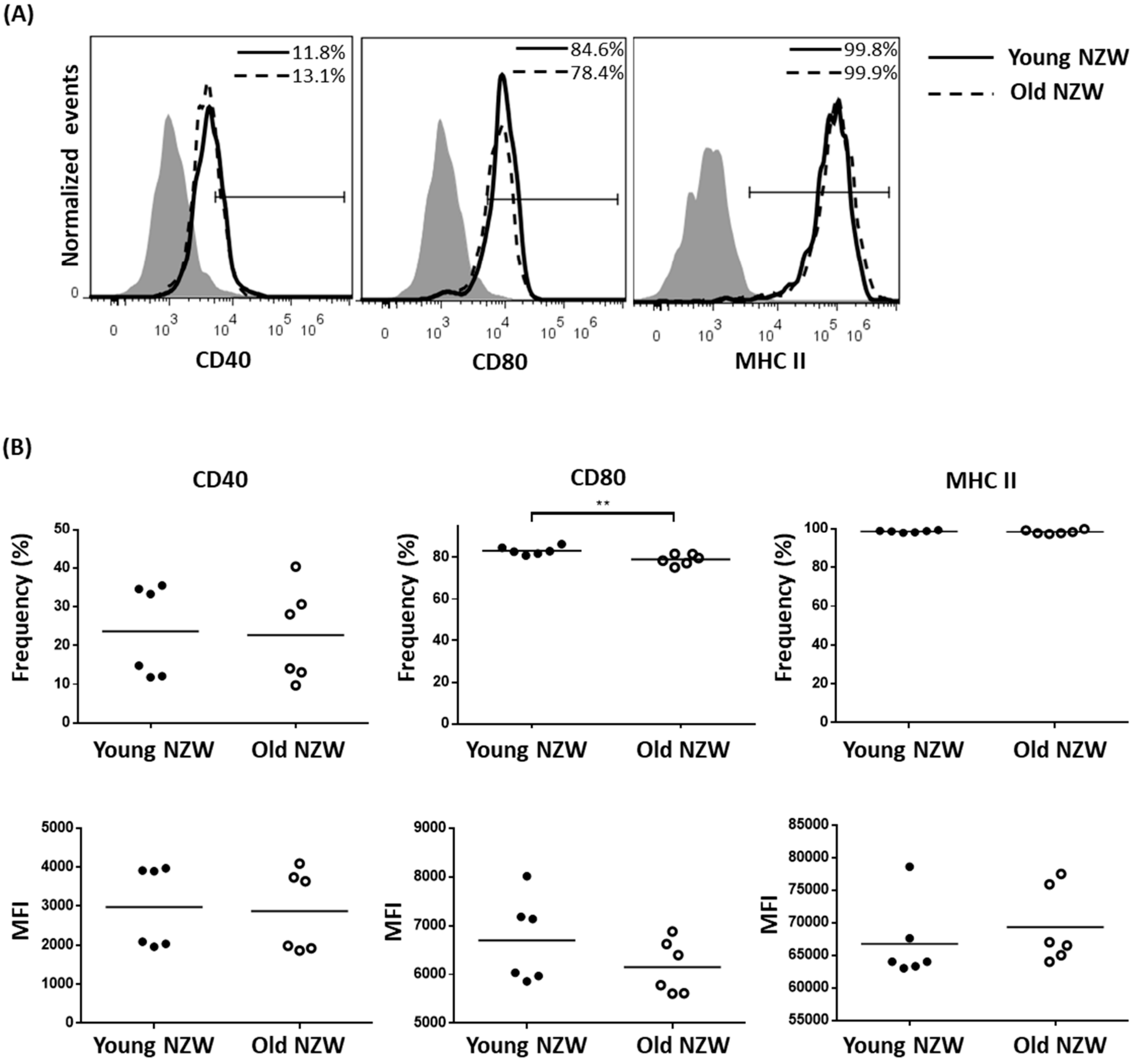

Supplementary Figure 3. Expression of co-stimulatory molecules and MHC II on CD11c^{hi}CD11b^{+} DCs from young and old NZW. Total splenocytes were stained with CD11c and CD11b together with the indicated activation marker. (A) Representative histograms showing the expression of the indicated markers on splenic CD11c^{hi}CD11b^{+} DCs in young and old NZW. Shade histogram represents the isotype control. (B) Summary plots showing expressions of CD40, CD80 and MHC II on CD11c^{hi}CD11b^{+} DCs in terms of frequency and mean fluorescence intensity (MFI) in young and old NZW. Each symbol represents an individual mouse and Student's t-test was used for statistical analysis (**p<0.01).

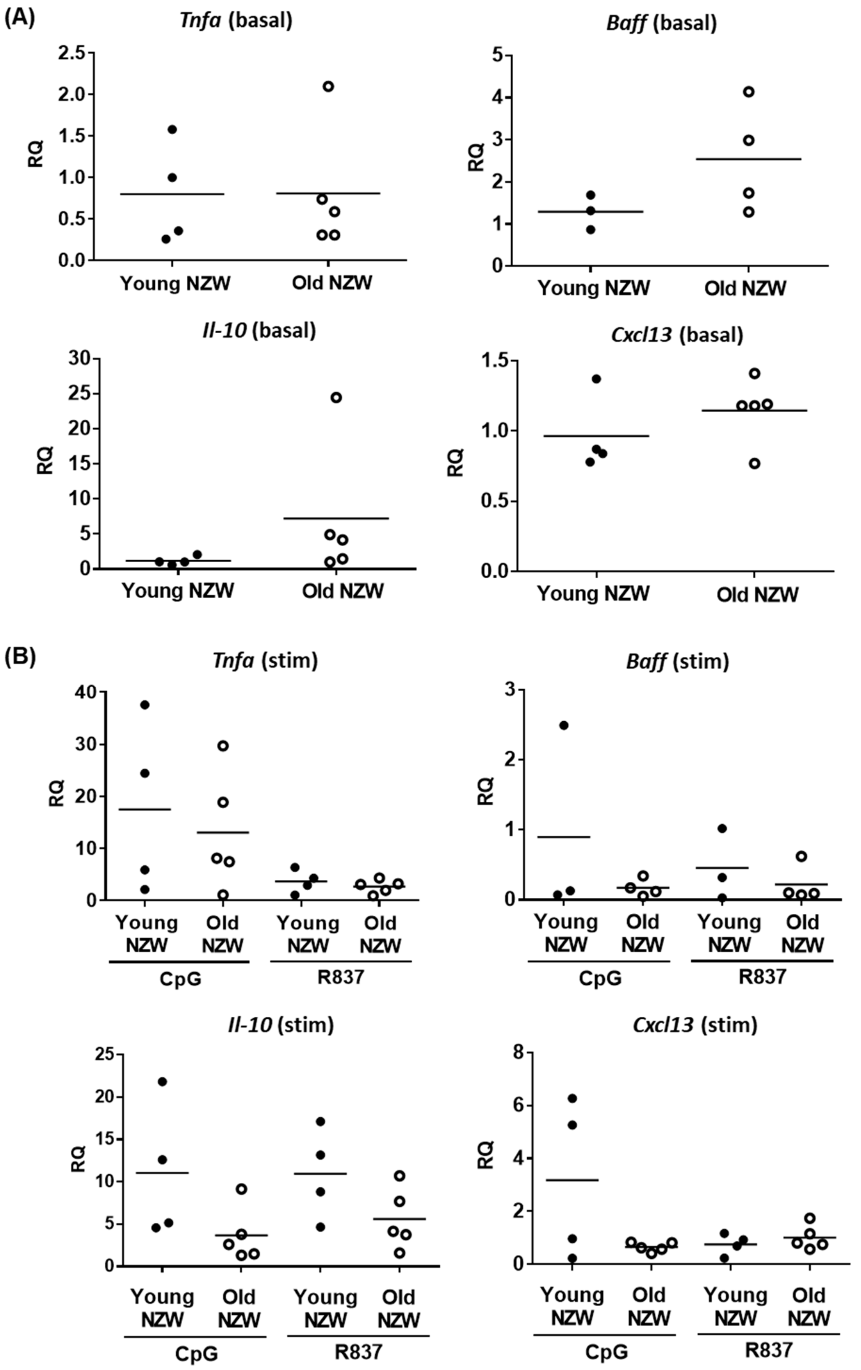

Supplementary Figure 4. Expressions of B cell and T cell stimulating cytokines and chemokines in young and old NZW. Splenic CD11c^{hi}CD11b^{+} DCs were FACS purified from NZW and mRNA expression was determined by qPCR. (**A**) Summary plots comparing the basal mRNA expression of *Tnfa*, *Baff*, *Il-10* and *Cxcl13* in Young and Old NZW mDCs. The mRNA expression was normalized with the house-keeping gene *β-actin*. The relative quantity (RQ) was the level of mRNA relative to unstimulated CD11c^{hi}CD11b^{+} DCs from young NZW. (**B**) For mRNA inductions, CD11c^{hi}CD11b^{+} DCs were stimulated for 5 h in the presence or absence of the TLR9 ligand CpG (1μM) or TLR7 ligand R837 (2.5μg/mL). The induction of mRNA is expressed as relative quantity (RQ) relative to un-stimulated CD11c^{hi}CD11b^{+} DCs of the respective group. For both (**A**) and (**B**), each symbol represents an individual mouse and Student's t-test was used for statistical analysis.

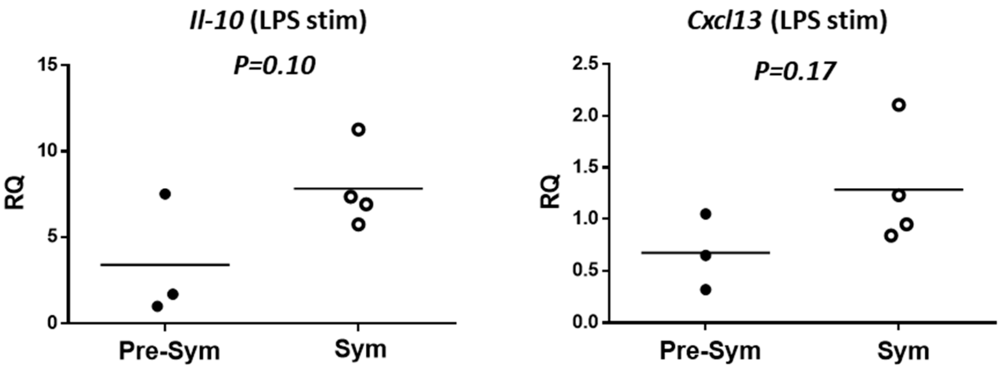

Supplementary Figure 5. CD11c^{hi}CD11b^{+} DCs from symptomatic BWF1 mice do not display heightened TLR4 responses. Splenic CD11c^{hi}CD11b^{+} cells from pre-symptomatic (Pre-sym) and symptomatic (Sym) BWF1 were FACS purified and stimulated with the TLR4 ligand LPS (5µg/mL) for 5 h. The mRNA expressions of *Il-10* and *Cxcl13* were determined by qPCR and was normalized with the house-keeping gene *β-actin*. The induction of mRNA is expressed as relative quantity (RQ) relative to un-stimulated cells of the respective group. Each symbol in summary plot represents an individual mouse from 3 independent experiments and Student's *t*-test was used for statistical analysis with the respective P value indicated in each graph.

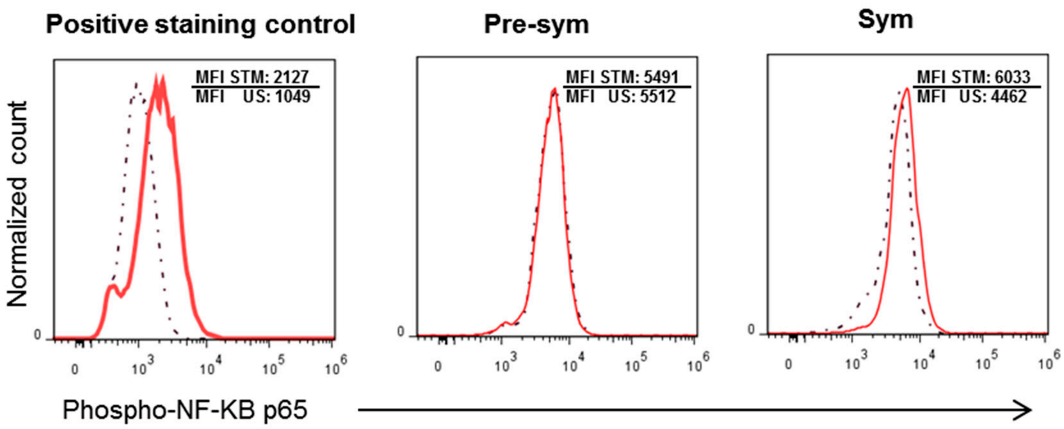

Supplementary Figure 6. CD11c^{hi}CD11b^{+} DCs from symptomatic BWF1 mice exhibit higher NF-κB p65 phosphorylation upon TLR9 stimulation. FACS-purified CD11c^{hi}CD11b^{+} DCs were stimulated with 5 μM CpG for 20 min and assayed for NF-κB activation by intracellular staining with an antibody against phosphorylated p65 NF-κB, followed by flow cytometry analysis. Each histogram represents staining result from purified cells from two mice of pre-symptomatic (Pre-sym) and symptomatic (Sym) BWF1. Dotted line represents expression of unstimulated (US) cells that cultured for the same period of time. Red solid line represents staining from stimulated (STM) cells. Number shown are mean fluorescence intensity (MFI). Positive staining control were prepared from staining of total splenocytes stimulated (STM) with PMA (100ng/mL) and ionomycin (500ng/mL) for 20 min or cultured with medium alone (US).

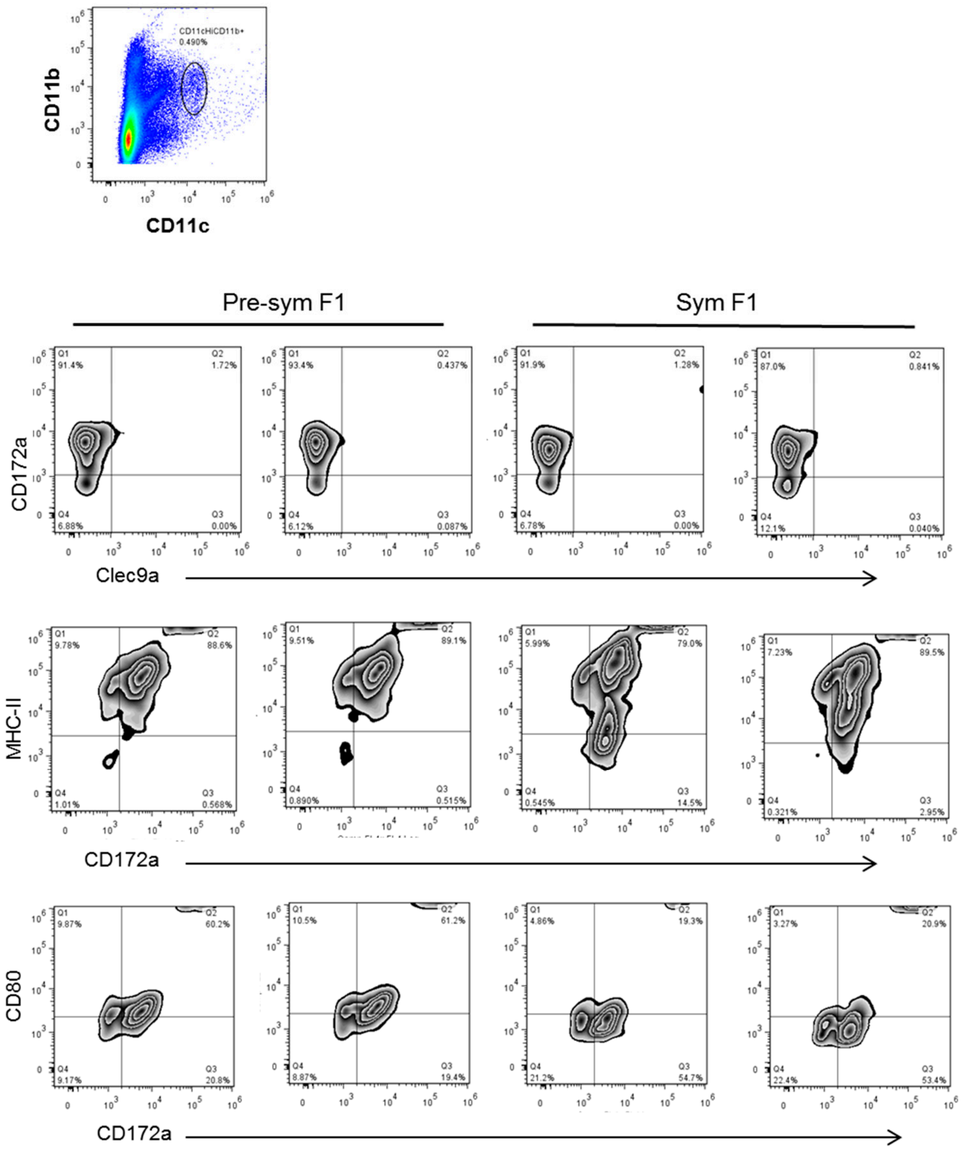

Supplementary Figure 7. CD11c^{hi}CD11b^{+} DCs consist of mainly cDC2 cells expressing CD172a. Splenocytes were stained with CD11c, CD11b, CD172a and Clec9a or MHC-II or CD80. Expressions of CD172a, Clec9a (top panel), MHC-II (middle panel) and CD80 (bottom panel) were determined on CD11c^{hi}CD11b^{+} gated cells from pre-symptomatic (Pre-sym) and symptomatic (Sym) BWF1 (F1). Each plot represents staining of individual mouse and two mice from each group were analyzed.
